# Mechanistic insights into methylene blue biodegradation by *Tetradesmus obliquus*: a multimodal approach using absorption, fluorescence, and square wave voltammetry

**DOI:** 10.1038/s41598-025-19675-3

**Published:** 2025-09-23

**Authors:** Merehan A. Talha, Hamdy E. Agwa, Amr M. Beltagi, Eithar El-Mohsnawy, Awatif S. Ali

**Affiliations:** 1 Botany and Microbiology Department, Faculty of Science, Kafrelsheikh University, Kafr El-Sheikh, 33516 Egypt; 2 Chemistry Department, Faculty of Science, Kafrelsheikh University, Kafr El-Sheikh, 33516 Egypt

**Keywords:** *Tetradesmus obliquus*, Methylene blue biodegradation, Absorption, Fluorescence, And square wave voltammetry, Biochemistry, Biological techniques, Biophysics, Microbiology, Physiology

## Abstract

Industrial dye wastewater production has surged alongside rapid industrial growth. This study evaluates the biodegradation of methylene blue (MB) by *Tetradesmus obliquus*, utilizing spectroscopical and electrochemical techniques to monitor effectiveness. Biodegradation model reveals conditions and pathways involved. *Tetradesmus obliquus* exhibits robust dye degradation, reducing absorbance at 664 nm significantly within days. MB concentrations of 2–4 mg/L were nearly completely degraded by day 6 and fully by day 9, with higher concentrations gradually reduced over 14 days. Square wave voltammetry showed full degradation of 0.2 mg/L MB by day 9. Fluorescence spectral analysis highlighted red-shifted emissions and decreased intensity due to MB presence. Growth patterns indicate *Tetradesmus obliquus* thrives post-MB adsorption, producing extracellular superoxide and hydrogen peroxide that convert MB to CO_2_, NO_2_, and H_2_O. Spectroscopic and electrochemical data confirm MB breakdown while boosting algal growth. This study demonstrates *Tetradesmus obliquus*’s efficacy in biodegrading MB from industrial textile wastewater, removing residual dye-bath additives and promoting environmental sustainability.

## Introduction

The worsening global shortage of freshwater is raising concerns about water pollution, particularly from chemical pollutants. Among the various sources of pollution, chemical pollutants pose a significant threat, including hazardous organic and inorganic substances that can severely harm aquatic ecosystems and threaten human health. Specifically, the annual industrial production of dye compounds is about 700,000 tons^1^. Organic dyes, synthetic chemicals used in the textiles, leather, and cosmetics industries, are persistent in the environment and can accumulate in the food chain, leading to long-term ecological and health risks^2^.

The widespread uses of dyes, coupled with their increasing annual production, have exacerbated the problem of dye-related wastewater pollution, becoming a major environmental concern. The bright colors of dyes obstruct light penetration, disrupting photosynthesis in aquatic ecosystems and contributing to increased chemical oxygen demand (COD). Furthermore, many dyes exhibit toxic, carcinogenic, and mutagenic properties, highlighting the urgent need for effective treatment of dye-saturated wastewater before its discharge into the environment^3–5^. Methylene blue (MB) is one such dye that poses considerable health and environmental risks due to its toxicity, carcinogenicity, and non-biodegradable nature. Its release into natural water sources becomes a serious threat to human health and aquatic life^6^.

The treatment of dye-contaminated wastewater presents a significant challenge due to the chemically stable structures and low biodegradability of synthetic dyes. Conventional methods—such as adsorption, photolysis, chemical oxidation and reduction, and electrochemical processes—often suffer from high operational costs, limited efficiency, and the risk of generating secondary pollutants. In response, recent research has increasingly focused on the application of microorganisms for the biodegradation and removal of synthetic dyes from aqueous solutions, industrial effluents, and wastewater streams. This process is relatively inexpensive, with low operational costs, and the end products of complete mineralization are non-toxic^7–9^.

Microbial degradation is considered an environmentally friendly, economical, and effective method for dye decolorization and degradation^10^. Various microorganisms have been reported for the decolorization of methylene blue (MB), including several fungi^11–13^, bacteria^7,14–17^. Additionally, fungi like *Phanerochaete chrysosporium* and green algae such as *Desmodesmus* spp. have also been identified for their ability to decolorize MB^6,18^.

Algae have garnered significant interest due to their local availability and cost-effectiveness. However, few studies have examined the efficiency of fresh algae in decolorizing dye effluents.

The objectives of this study were to evaluate the effectiveness of *Tetradesmus obliquus* in biodegrading the dye methylene blue (MB), monitor this process using two different sensitive spectroscopic and electrochemical techniques, and elucidate the possible mechanism of this process.

## Materials and methods

### Cultivation and growth condition

*Tetradesmus obliquus* was cultivated in triplicate within 1000 ml Erlenmeyer flasks, each contained 500 ml of sterilized Bold Basal medium (BB medium) as described by Nichols & Bold^19^. For industrial wastewater treatment, wastewater was sourced from Seginy Company in Mahalla Kubra, Egypt. Bold Basal medium stock solutions were added to the wastewater prior to inoculation with an aged algal suspension. A 10 ml aliquot of a 16-day-old algal suspension was inoculated into the fresh medium, ensuring an initial optical density of 0.05 at OD 680 nm, following the methodology of El-Mohsnawy et al.^20^. The cultures were illuminated with tubular white fluorescent lamps, and the light intensity was adjusted at 80 µmol/m²/s and monitored by UNI-T UT383. The flasks were incubated at 25 ± 1 °C with gentle stirring facilitated by a sterilized air pump stream. Algal growth was monitored daily by measuring optical density at 680 nm using a Visible Spectrophotometer (Jasco V-730). The algal biomass was harvested during the logarithmic phase, which was reached after 14 days of cultivation.

### Estimation of the methylene blue biodegradation

#### Using spectroscopical method

The spectroscopic measurements were performed using a spectrophotometer (Jasco V-730 UV-Visible Spectrophotometer) through an absorption scan 400–700 nm. Algal cultures were inoculated with MB dye, reaching final MB concentrations of 2, 4, 8, 16, 20, and 24 mg/L in addition to a control (free of dye). At 0, 4, 6, 9, 11 and 14 day, algal cultures were centrifuged at 5000 g.Supernatant free cells was spectroscopically analyzed using absorption scan 400–700 nm at room temperature.

#### Using voltametric method

The voltammetric measurements were performed using a PalmSens 4 Potentiostat/Galvanostat/Impedance Analyzer (PalmSens BV, Randhoeve 221, 3995 GA Houten, The Netherlands), which was computer-controlled. The PSTrace 5 software version 5.9 (PalmSens BV) was utilized for controlling the instrument. A micro-voltammetric cell (PAR Model K0262) consisting of a carbon paste electrode body (BAS model MF-2010), an Ag/AgCl/3 M KCl reference electrode (PAR K0265), and a platinum wire counter electrode (PAR model K0266) was used. The body of the carbon paste electrode was a Teflon rod with an end cavity of 3 mm diameter and 1 mm deep bored at one end for paste filling. Contact was made with a copper wire through the center of the Teflon rod.The de-ionized water used throughout the present work was obtained from a (Milli-Q^®^ Model Gradient A10, MILLIPORE, France) Deionizer connected to a Hamilton-Aqua Matic bidistillation water system (Hamilton Laboratory Glass LTD, Kent, UK). All experiments have been conducted at room temperature.

##### Chemicals and reagents

Graphite powder, CH_3_COOH and CH_3_COONa were purchased from Sigma-Aldrich. All the chemicals used in this work were of analytical reagent grades and have been used as received without further purification. Acetate buffer solution was prepared in de-ionized water and was used as supporting electrolytes.

##### Preparation of the carbon paste electrode (CPE)

The carbon paste was prepared by thoroughly hand mixing 5 g of graphite powder (1–2 μm) with 1.8 ml of Nujol (Sigma, *d* = 0.84 g/ml) oil in an agate mortar with pestle. The paste was then packed into the cavity of the electrode, and its surface was manually smoothed by polishing on a clean tracing paper.

##### General analytical procedures

A 10-ml volume of acetate buffer solution (pH = 5.0) was introduced into the micro-electrolysis cell, and the smoothed CPE was then immersed in the supporting electrolyte, and several cyclic voltammetry sweeps were applied to obtain a low background current. After that, aliquots of the analyte were introduced into the electrolysis cell, and the voltammogram was then recorded by scanning the potential from 0.0 to -0.07 V using the square-wave potential waveform. After each measurement, the CPE was carefully removed and re-constructed. The optimal square wave voltammetry (SWV) instrumental conditions were: frequency = 10 Hz, potential step = 10 mV, and amplitude = 25 mV.

### Spectrofluorometric analysis

An FP-8650 NIR spectrofluorometer was used to perform fluorimetric measurements. For sample preparation, 50,100,150,200,250 µl of *Tetradesmus obliquus* were mixed with 1.5 ml of 24 mg/L MB dye. Pure MB dye in algal medium and pure *Tetradesmus obliquus* were used as control. The samples were excited using monochromatic light at 440–600 nm, with a bandwidth of 2.5 nm, an emission bandwidth of 5 nm, and a scan rate of 1 nm/s. Emission spectra in a range of 600–800 nm were recorded. The corresponding algal dried biomass for the used samples were 0.115, 0.23,0.345,0.46 and 0.575 mg.

The samples were excited using monochromatic light at 440–600 nm, with a bandwidth of 2.5 nm, an emission bandwidth of 5 nm, and a scan rate of 1 nm/s.

## Results

**Fig. 1:**
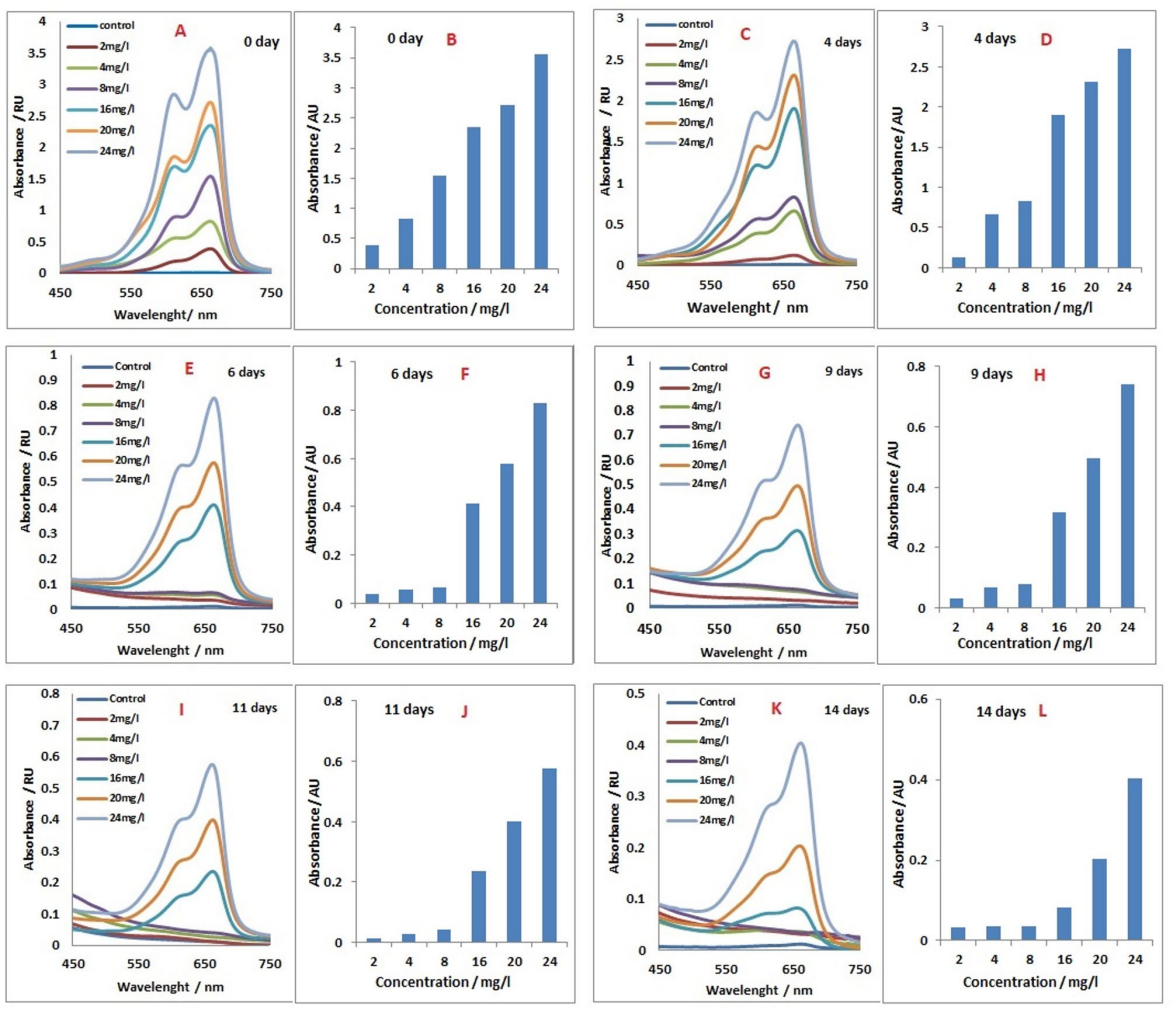
Absorption spectral analysis 450–750 nm (**A**, **C**, **E**, **G**, **I**, **K**), *Tetradesmus obliquus* culture filtrates grown on different methylene blue concentrations at different incubation periods (0, 4, 6, 9, 11 and 14 days). Biodegradation rate of different concentrations of methylene blue by incubation in *Tetradesmus obliquus* cultures (**B**, **D**, **F**, **H**, **J**, **L**).

*Tetradesmus obliquus* showed strong biodegradation ability of the methylene blue (MB) dye. The maximum absorbance of the dye was detected at 664 nm. At day zero time, a linear relationship between concentration and absorbance was identified at 0 day (Fig. 1, A and B). Almost the same reduction of absorption was observed for all concentrations after 4 days (Fig. 1, C and D). After 6 days, the concentrations of 2 and 4 mg/L were almost completely degraded (Fig. 1, E and F). After 9 days, the concentrations of 2, 4, and 8 mg/L were completely degraded (Fig. 1, G and H). Dye absorbance was gradually decreased to be 0.39 for 24 mg/L (Fig. 1, K and L). Here, it is clear that a gradual reduction in absorbance of concentrations 16, 20, and 24 mg/L was recorded from 2.42, 2.5, and 3.5 at time zero to be 0.07, 0.18, and 0.39, respectively, after 14 days.

**Fig. 2:**
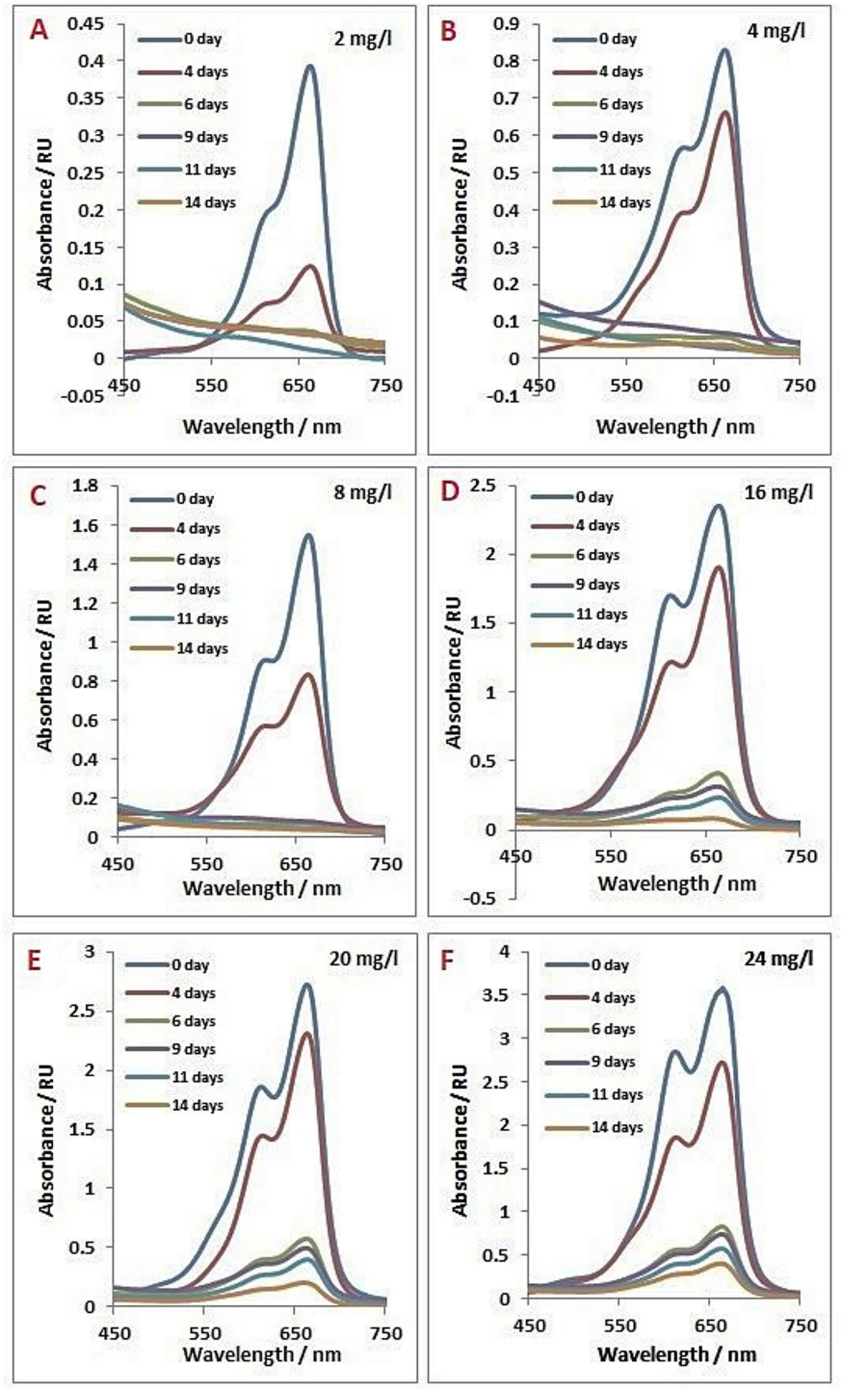
Absorption spectral changes of *Tetradesmus obliquus* culture filtrates 0–14 days grown on different methylene blue concentrations.

**Fig. 3:**
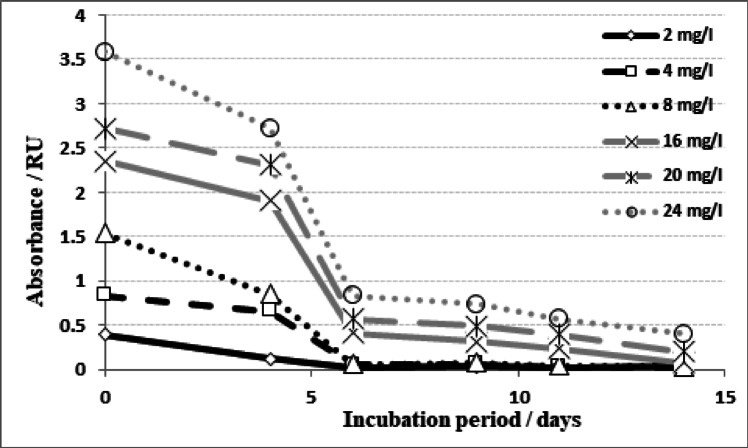
Change in absorbance of different concentrations of methylene blue (2, 4, 8, 16, 20 and 24 mg/L) during cultivation on *Tetradesmus obliquus* culture for 0–14 days.

Absorption spectra of algal filtrate containing MB dye concentrations through 14 days incubation with ***Tetradesmus obliquus*** exhibited the changing occurred in dye concentration by the time, as well as the biodegradation rate of dye. At low MB dye concentration, 2 mg/L, alga was able to degradate about 75% of dye within 4 days and reached complete degradation within 6 days. Also, alga did not show lag phase during the biodegradation of dye that shown in other treatments. Almost the same behavior was obtained by the other treatments, where disappearing rate of MB showed low rate till the 4th day followed by high slope till the 6th day. At the 6th day, most of dyes disappeared. After the 6th day, the rate of degradation returned to be slow again. MB dye exhibited complete degradation at concentrations 2, 4 and 8 mg/L, while the other concentrations remained traces even after 14 days of incubation periods (Figs. 2 and 3).

**Fig. 4:**
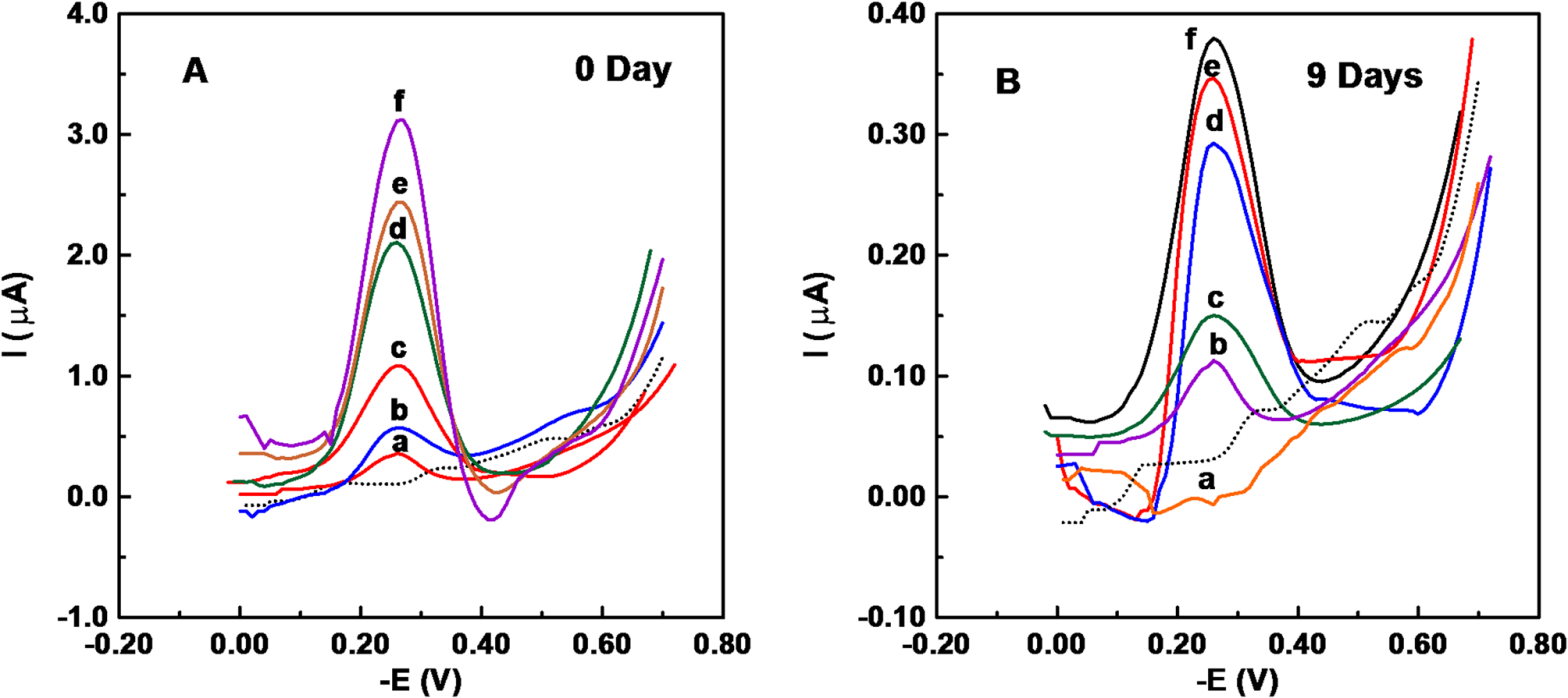
Square wave voltammograms of different MB concentrations in acetate buffer solution of pH 5.0 at a carbon paste electrode applying a frequency of 10 Hz, potential step of 10 mV and amplitude of 25 mV; (a) 0.2, (b) 0.4, (c) 0.8, (d) 1.6, (e) 2.0 and (f) 2.4 mg/L MB. Dotted curve represents the blank acetate buffer solution. A and B are for 0 and 9 days, consequently.

A single well-defined cathodic peak was obtained at – 0.273 V (vs. Ag/AgCl) onto the carbon paste electrode (CPE). Voltammograms of MB onto the CPE were performed by varying the square wave parameters within the designated ranges: frequency (*f* = 2–20 Hz), potential step (Δ*E*_s_ = 2–10 mV), and pulse amplitude (*E*_sw_ = 25–50 mV). Frequency *f* = 10 Hz, potential step (Δ*E*_s_ = 10 mV, and pulse amplitude *E*_sw_ = 25 mV were determined to be the optimal operating parameters in order to attain the best peak morphology, peak current intensity and minimum background. Figure 4(A) shows the square-wave cathodic voltammograms that were acquired for increasing MB concentrations applying the optimal instrumental conditions onto the CPE. The peak current intensity exhibited a linear increase with increasing MB content within the dynamic range of 0.05–40 mg/L, as described by the equation: *i*_p_ (µA) = 1.1697 C ± 0.056 (mg/L) + 0.0572 ± 0.005 (r^2^ = 0.9977).

Square-wave voltammograms of solutions of different concentrations of MB following biodegradation by *Tetradesmus obliquus* (incubation for 9 days) exhibited substantial decline in peak current intensities compared with those of identical concentrations without degradation (Fig. 4A& B). The initial 2.0 mg/L concentration was totally broken down, and the voltammogram showed no peak. The de-colorization and adsorption degradation efficiency of different concentrations (2.0–24 mg/L) have been calculated as:

$$\:E\left(\%\right)=\left(\frac{{C}_{0}-\:{C}_{t}}{{C}_{0}}\right)\times\:100$$

As shown in Table 1, the degradation efficiency is 100% for concentration of 2.0 mg/L MB and decreased gradually to almost leveled-off at concentration levels of 16–24 mg/L (89.55–88.56%). These results confirmed the efficiency of *Tetradesmus obliquus* for the biodegradation of MB, even at high dye concentration levels.

**Table 1:** Concentration values of MB before and after biodegradation, with the degradation efficiency.

Concentration of [MB] (mg/L)		Degradation efficiency (E%)
Before biodegradation (Taken–Initial)	After biodegradation (at 9th day of incubation)	
2.0	–	100
4.0	0.092	97.70
8.0	0.644	91.95
16	1.830	88.56
20	2.090	89.55
24	2.727	88.64

**Fig. 5:**
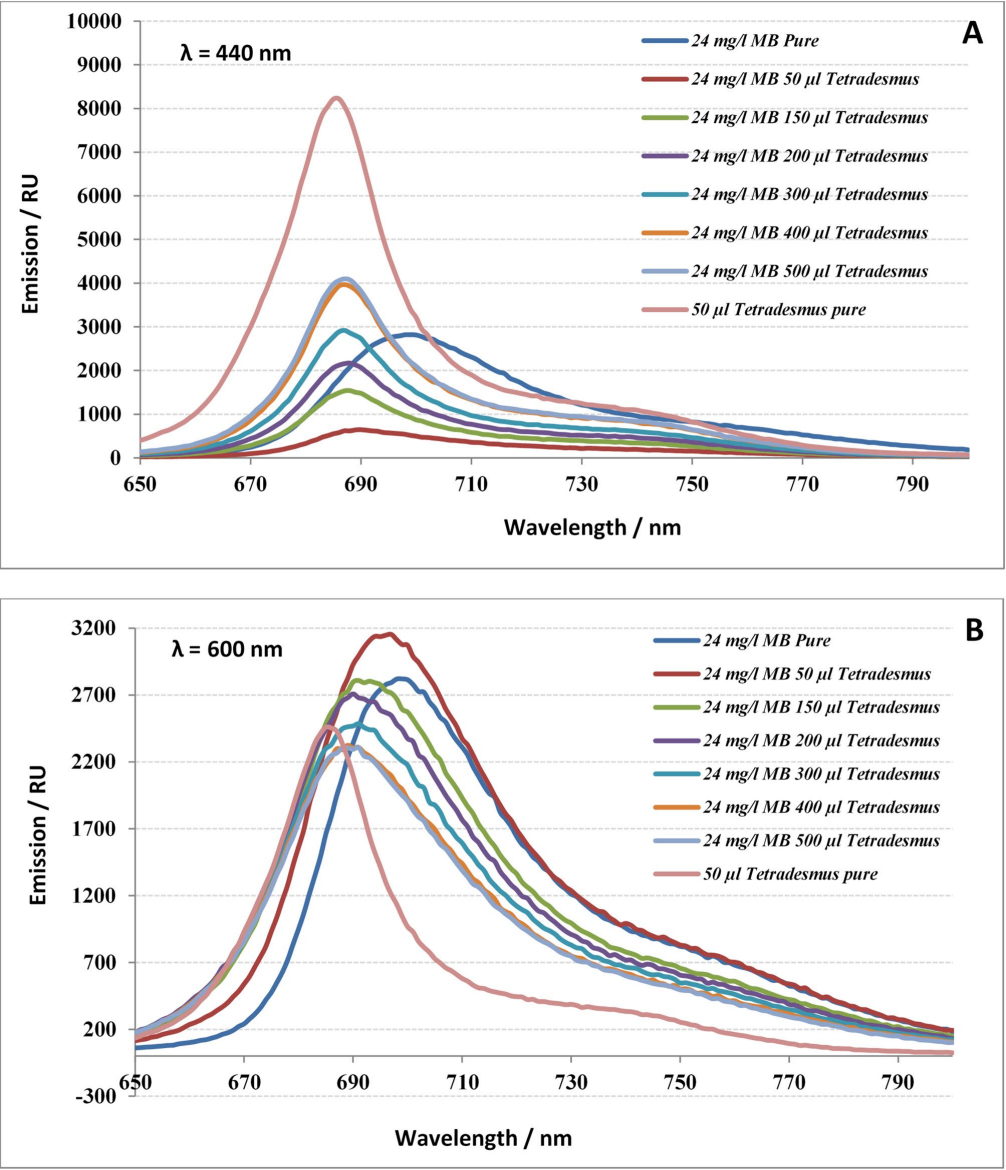
Fluorescence emission spectra of 24 mg/L MB dye mixed with serial biomass concentrations of *Tetradesmus obliquus.* a: at λ = 440 nm and b: λ = 600 nm.

The fluorescence emission spectral analysis of *Tetradesmus obliquus*, exited at 440 nm, displayed significant variations influenced by the presence of methylene blue (MB) dye. At pure *Tetradesmus obliquus* samples the observed emission was 8222 at 686 nm. Emission was red-shifted to 690 nm, broader and reduced to be 644by adding 24 mg/L MB dye. When more algal cells were added to be 150, 200, 300, 400 and 500 µL, peaks shifted to blue region and peaks heights were 1540, 2170, 2920, 3968 and 4098, respectively. MB pure dye exhibited maximum emission, 2820 at 699 nm (Fig. 5a). Almost the same behavior was noticed in case of exited the sample by 600 nm monochromatic light. Pure MB sample exhibited the maximum emission, 2820 at 699 nm. By adding 50, 150, 200, 300, 400, 500 µl, emission of mixtures was red-shifted to be 697, 691, 690, 690, 689 and 688 nm, respectively. Pure *Tetradesmus obliquus* sample exhibited maximum emission spectra at 688 nm with the highest value of 2338 (Fig. 5b).

**Fig. 6:**
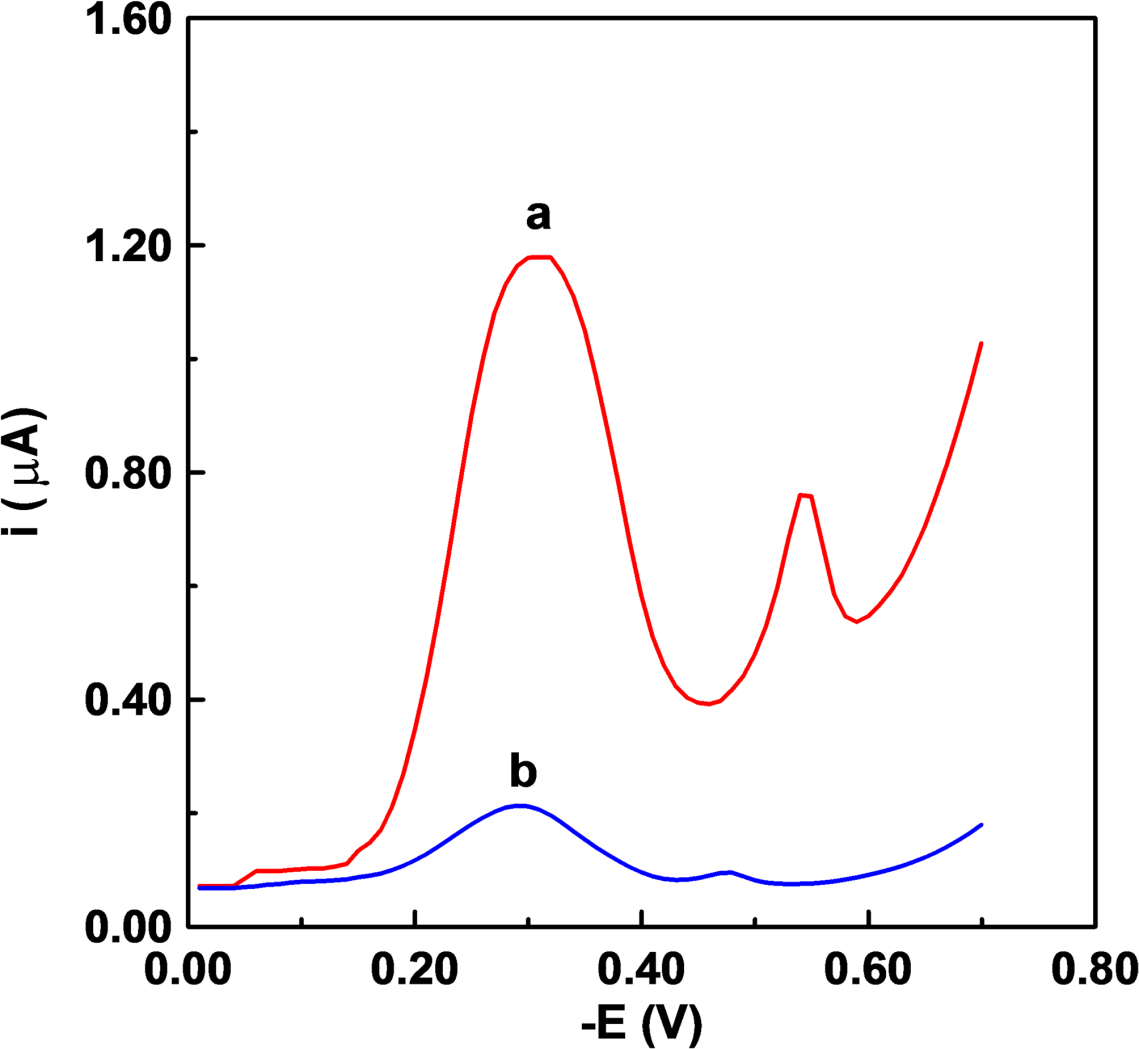
Square wave voltammograms of a real industrial wastewater sample containing MB in acetate buffer solution of pH 5.0 at a carbon paste electrode applying a frequency of 10 Hz, potential step of 10 mV and amplitude of 25 mV; (a) before degradation and (b) after biodegradation for 9 days.

**Fig. 7:**
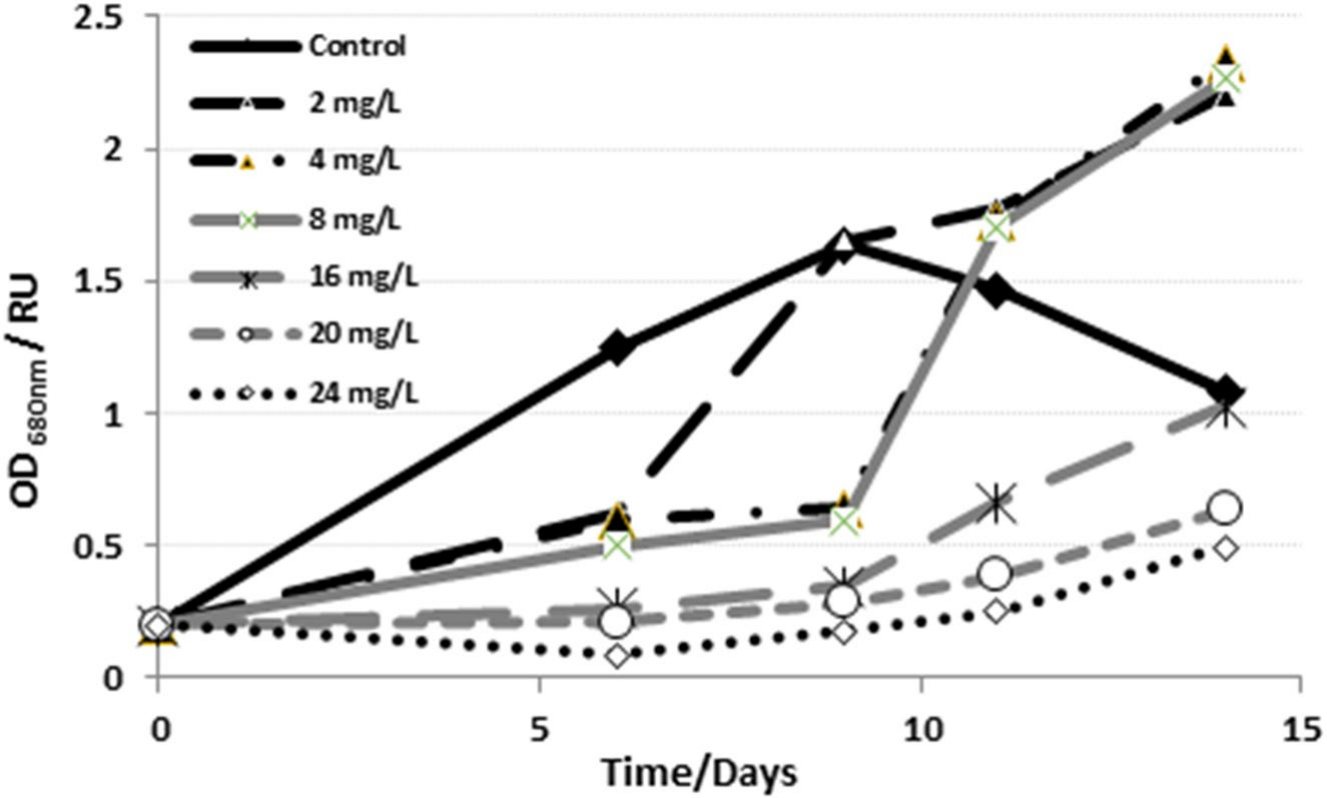
Growth curve of *Tetradesmus obliquus* incubated with different concentrations of MB dye.

The proposed biodegradation approach utilizing *Tetradesmus obliquus* was effectively implemented for the treatment of a real industrial wastewater sample from the textile dyeing process, primarily containing MB, red reactive azo-dyes, and NaCl (0.0345–0.052 M) as a residual dye-bath additives. The SW voltammogram of the wastewater sample exhibited a peak corresponding to MB at − 0.306 V, alongside a peak at − 0.544 V, which may be attributed to the reduction of azo-dyes present in the sample (Fig. 6a). The voltammogram of the wastewater sample exhibited a significant decrease in the peak current intensity of both peaks (Fig. 6b). The initial concentration of MB in the wastewater sample was determined to be 9.730 mg/L, whereas the final concentration after 9 days of biodegradation was measured at 1.161 mg/L, resulting in a degradation efficiency of 88.07%. These results validated the efficacy of the proposed degradation process for use in real industrial wastewater samples. Growth pattern of ***Tetradesmus obliquus*** in pure culture and different concentrations of MB dye at 0, 6, 9, 11 and 14 days. Alga in pure culture showed the fastest growth rate till the maximum absorption, OD_680_ 1.65, at the 9th day and turned down afterwards reaching OD_680_ 1.02 at the 14th day. By adding 2 mg/L MB, the growth rate reduced till the 6th day reaching OD_680_ 0.598, after that it enhanced to reach OD_680_ 1.652 at the 9th day and continued raising to reach OD_680_ 2.2 at the 14th day. Almost the same behavior was obtained in case of adding 4 and 8 mg/L MB, where the rate of growth was slow till the 9th day, after that they started raising in growth rate to reach OD_680_ 2.33 and 2.26 for 4 and 8 mg/L, respectively. By adding high MB dye concentration, 20 and 24 mg/L, the growth of ***Tetradesmus obliquus*** reduced to be OD_680_ 0.21 and 0.09 at the 6th day after that they started raising in slow rate to reach OD_680_ 0.635 and 0.495 for 20 and 24 mg/L MB dye, respectively (Fig. 7).

## Discussion

Due to the extensive use of dyes and increasing annual production, dye wastewater pollution has become a major environmental issue, attracting significant attention^21^. The high chromaticity of dyes not only affects the photosynthesis of aquatic systems but also increases chemical oxygen demand (COD)^22^; additionally, dyes are toxic, carcinogenic, and mutagenic^23^. Therefore, dye-containing wastewater must be treated before being discharged into the environment.

The obtained results reveal that *Tetradesmus obliquus* efficiently decolorized methylene blue (MB) dye, with different concentrations showing almost complete degradation within 6 to 14 days, and absorbance at 664 nm progressively decreasing over time, demonstrating the algae’s strong biodegradation ability. Square wave voltammetry indicated significant biodegradation of MB dye by *Tetradesmus obliquus* over 9 days, with currents reducing across various concentrations and complete degradation of 0.2 mg/L MB, while higher concentrations showed varying degradation percentages. *Desmodesmus* also efficiently decolorized MB from aqueous solutions with up to 98.3% dye removal, though effectiveness depended on factors such as initial dye concentration, contact time, and immobilization^7,18^.

Fluorescence emission spectral analysis of *Tetradesmus obliquus* excited at 440 nm showed variations due to the presence of MB dye, with emissions red-shifting and decreasing in intensity, while pure MB dye exhibited maximum emission at 699 nm. Mixed samples exhibited shifted emissions with varying intensities depending on the amount of algae added. Green algae emit fluorescence primarily from chlorophyll a at around 680 nm when excited by red or blue actinic light, while MB emits in the red region when excited by blue or red light. When both are present together, MB can quench chlorophyll fluorescence, resulting in decreased overall fluorescence intensity and interference with measurements^24,25^.

The growth pattern of *Tetradesmus obliquus* in pure culture and various concentrations of MB dye showed that the algae grew fastest in pure culture. Growth was initially reduced in the presence of MB, followed by enhancement over time, with higher MB concentrations resulting in slower initial growth but eventual increases, with maximum absorbance values varying across concentrations. MB significantly inhibited the growth of microalgae, including Chlorophytes, in a concentration-dependent manner, affecting metabolic activities and disrupting normal cellular functions^18,23,26^. Because of its sensitivity and speed^27^, the square-wave voltammetry technique was selected to optimize an ultra-sensitive analytical method for the monitoring of MB dye both before and after bioremediation. Preliminary experiments for the voltammetric determination of MB in various supporting electrolytes (Britton-Robinson buffer of pH 2–11 and acetate buffer of pH 4.2–6.0) indicated that the highest peak current intensity was achieved in an acetate buffer of pH 5.0. The growth phase of *Tetradesmus obliquus* was prolonged the lag phase when treated with MB, with the duration positively correlated to the dye concentration (Fig. 7). During the initial growth stage, the high dye concentration relative to the algal inoculum acted as a mask, screening some incident photons and extending the lag phase. However, as the algae adapted and underwent enzymatic modifications, it began to grow efficiently during the exponential phase^28^. The main reason for enhancing *Tetradesmus obliquus* growth is the biodegradation of MB due to adsorption, where MB molecules adhere to the algal cell surface through electrostatic interactions. Algal cell surfaces typically have negatively charged components, such as cell walls and membranes, which attract positively charged MB molecules. The production of extracellular superoxide and hydrogen peroxide by the algae degrades MB into CO_2_, NO_2_, and H_2_O [7,17–18,29–30]. Various techniques, including adsorption, phytoremediation, coagulation, ultrafiltration, nanofiltration, electrocoagulation, microwave treatment, and biodegradation, have been employed to remove MB and textile dyes from industrial wastewater. However, MB’s thermal and light stability, along with its non-biodegradability, presents challenges for complete degradation into smaller inorganic molecules. Each method offers unique benefits and limitations in terms of cost, efficiency, and environmental impact^6^. Chemical MB treatment exhibited so dangerous artifact, where photocatalytic degradation of methylene blue (MB) involves reactive radicals like h^+^, •OH, and •O_2_^−^, which oxidize MB into CO_2_, H_2_O, and inorganic ions such as nitrate (NO_3_^−^), ammonium (NH_4_^+^), and sulfate (SO_4_^2−^)^31^. Aquatic creatures are so sensitive to different heavy metals. The metals and heavy metals commonly used in the photocatalytic degradation of methylene blue (MB) are essential for enhancing its degradation efficiency. Examples include Zinc (Zn), utilized in ZnO and Zn Sphotocatalysts^32^, Titanium (Ti), found in TiO_2_^33^, and Iron (Fe), present in hematite (Fe_2_O_3_)^34^. Copper (Cu) is frequently incorporated in metal vanadates or doped into ZnO [35–36], while Bismuth (Bi) is employed in bismuth vanadates (BiVO_4_), Nickel (Ni) also appears in nickel vanadates and Plasmonic metals such as Silver (Ag), Gold (Au), and Platinum (Pt) improve photocatalytic performance through enhanced light absorption and charge separation^6^. Present work, biological treatment using *Tetradesmus obliquus*, exhibited clean pathway where it is not only reduce the toxic compounds but also consuming the products. Using three different analytical approaches showed an obvious data concerning the absence of intermediate toxic compounds that reported in chemical methods. Detection of harmful intermediates by chemical treatment of MB is confirmed by the reduction of MB’s absorption peak^37^ and UV–vis spectra analysis^38^. Azure dyes and aniline are identified as intermediate by-products formed through demethylation of MB^39^. Active radicals degrade MB’s molecular bonds, such as N–CH3, C–N, and C–S, converting them into stable inorganic molecules^40^.

Some microalgae can use azo dyes as a mixotrophic nutrient source, metabolizing the dyes to detoxify them and utilize the byproducts for growth. Microalgae remove dyes through mechanisms like biosorption, bioconversion, and biodegradation. Laccase glycoproteins play a key role in breaking down many industrial dyes and phenols^41^. Algae degrade these dyes and incorporate them into their growth process, helping reduce eutrophication in aquatic ecosystems^42^.The biodegradation approach using *Tetradesmus obliquus* successfully treated industrial wastewater from the textile dyeing process, effectively addressing contaminants such as methylene blue (MB), red reactive azo-dyes, and residual dye-bath additives like NaCl^43^.

The results obtained and the proposed model elucidated the following facts: the absence of lag in adaptation at low MB concentrations, the saturation impact at elevated concentrations (e.g., 16–24 mg/L), and the growth-associated degradation as evidenced by OD_680_ trends. Consequently, the Monod-based model appears to be the most appropriate kinetic model, as Tetradesmus obliquus demonstrates vigorous growth and effective adaptation to fluctuating MB concentrations throughout time.

In contrast^44^, clarified that the first-order models can misrepresent biodegradation when substrate concentrations exceed the half-saturation constant (Ks). Monod kinetics better reflect the biological reality in such cases.

**Fig. 8:**
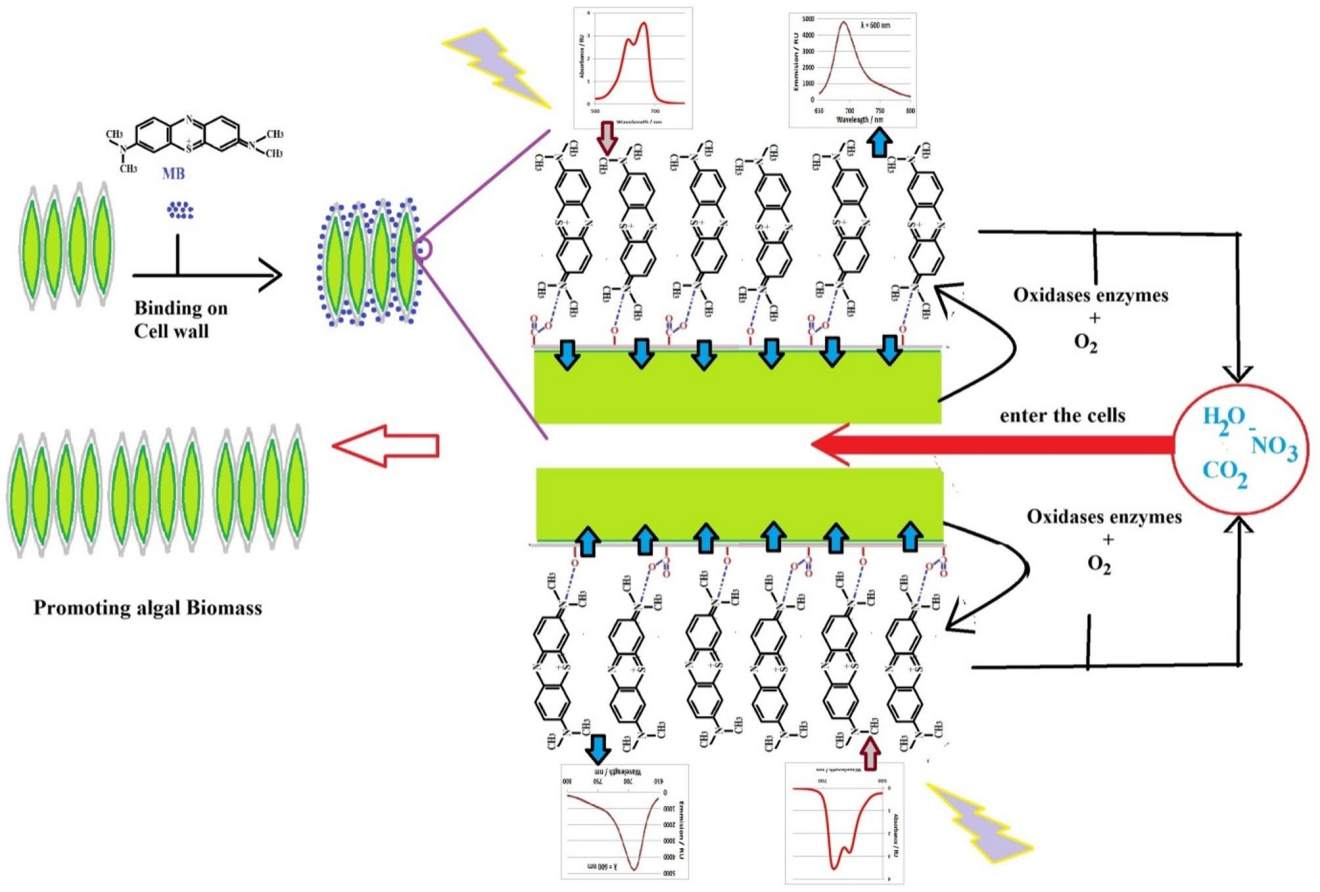
Suggested model for biodegradation MB and biomass enhancing mechanism of *Tetradesmus obliquus* .

Figure 8 illustrates the possible mechanism by which *Tetradesmus obliquus* contributes to the eco-friendly biodegradation of MB, a serious pollutant affecting aquacultures. Based on our results and the reported literatures applied on more related organisms, the degradation pathway starts with biosorption. It is performed via passive binding of dye molecules to algal cell walls via functional groups (e.g., hydroxyl, carboxyl, sulfate), which is considered a Langmuir adsorption isotherm^45^. After adsorption comes exoenzymatic biodegradation by *Tetradesmus obliquus.* Active breakdown of dye molecules by algal enzymes such as laccases and peroxidases can be monitored through UV-vis spectral analysis, fluorescent analysis, and Square wave voltammetry of current work, as well as FTIR^46^, where the spectral shifts confirm chemical transformation of MB after algal treatment. MB binds to the surface of *Tetradesmus obliquus* walls, competing with Chlorophyll *a* and blocking light from reaching the photosynthetic apparatus. During this period, *Tetradesmus obliquus* produces extracellular superoxide and hydrogen peroxide, degrading MB into CO_2_, NO_2_, and H_2_O. The final step is photodegradation, where *Tetradesmus obliquus* enhances the photodegradation by producing reactive oxygen species that resulted from photosynthesis process under light exposure^18^.

## Conclusion

Current study highlights the potential cost-effective biodegradation method of methylene blue (MB) by *Tetradesmus obliquus* at various concentration levels. Spectrophotometric analysis revealed a steady decrease in dye uptake over time, with complete degradation achieved within 9 days at 2, 4, and 8 mg/L. Even at higher concentrations (16–24 mg/L), a significant reduction was observed, reaching an efficiency of up to 88.56% after 14 days. Square wave voltammetry further confirmed the degradation process, showing reduced peak current intensities after incubation, with optimal electrochemical parameters that improved detection sensitivity. Fluorescence emission spectra exhibited significant spectral shifts and intensity changes upon exposure to MB, suggesting interactions between the dye molecules and algal cells. The growth dynamics of *T. obliquus* were also influenced by MB concentration, with low concentrations promoting recovery and enhancing biomass accumulation, while higher concentrations initially suppressed growth before gradual adaptation. Notably, the biodegradation strategy proved effective in treating real-life industrial wastewater containing MB, achieving an 88.07% reduction in MB concentration. These results underscore the ecological and practical suitability of *T. obliquus* as an effective and eco-friendly bioremediation agent for dye-polluted industrial wastewater. The integration of spectroscopic, voltammetric, and growth analyses provides a comprehensive understanding of the degradation mechanisms and supports the application of microalgae in environmental detoxification.

## Data Availability

The datasets used and/or analyzed during the current study available from the corresponding author on reasonable request.
